# Towards Intense THz Spectroscopy on Water: Characterization of Optical Rectification by GaP, OH1, and DSTMS at OPA Wavelengths

**DOI:** 10.3390/ma13061311

**Published:** 2020-03-13

**Authors:** Fabio Novelli, Biswajit Guchhait, Martina Havenith

**Affiliations:** 1 Department of Physical Chemistry II, Ruhr University Bochum, 44801 Bochum, Germany; biswajit.guchhait@snu.edu.in; 2 Department of Chemistry, School of Natural Sciences, Shiv Nadar University, Greater Noida, Uttar Pradesh 201314, India

**Keywords:** terahertz, water, solvation, hydration, non-linear optics, spectroscopy

## Abstract

Water is the most prominent solvent. The unique properties of water are rooted in the dynamical hydrogen-bonded network. While TeraHertz (THz) radiation can probe directly the collective molecular network, several open issues remain about the interpretation of these highly anharmonic, coupled bands. In order to address this problem, we need intense THz radiation able to drive the liquid into the nonlinear response regime. Firstly, in this study, we summarize the available brilliant THz sources and compare their emission properties. Secondly, we characterize the THz emission by Gallium Phosphide (GaP), 2–{3–(4–hydroxystyryl)–5,5–dimethylcyclohex–2–enylidene}malononitrile (OH1), and 4–N,N–dimethylamino–4′–N′–methyl–stilbazolium 2,4,6–trimethylbenzenesulfonate (DSTMS) crystals pumped by an amplified near-infrared (NIR) laser with tunable wavelength. We found that both OH1 as well as DSTMS could convert NIR laser radiation between 1200 and 2500 nm into THz radiation with high efficiency (> 2 × 10^−4^), resulting in THz peak fields exceeding 0.1 MV/cm for modest pump excitation (~ mJ/cm^2^). DSTMS emits the broadest spectrum, covering the entire bandwidth of our detector from ca. 0.5 to ~7 THz, also at a laser wavelength of 2100 nm. Future improvements will require handling the photothermal damage of these delicate organic crystals, and increasing the THz frequency.

## 1. Introduction

Terahertz (THz) radiation probes the collective intermolecular modes of hydrogen-bonded water molecules from ~0.2 to ~20 THz [1]. These modes [1,2,3,4,5] represent the microscopic solvent coordinate for proton transfer [6], are involved in bio-reactions like drug intercalation into DNA [7,8] and, according to molecular dynamics simulations [9], are the drive of the structural rearrangements of the molecular network. The collective transient dynamics of the hydrogen-bonded network dictate the unique solvation properties of water, and are at the origin of the puzzling physical and chemical properties of this special liquid [10,11,12]. However, pivotal issues remain about the interpretation of these THz features [13]. For example, mapping how the energy dissipates upon excitation of a low frequency mode could allow a deeper understanding and, possibly, engineering of solvation processes [14,15].

**Figure 1:**
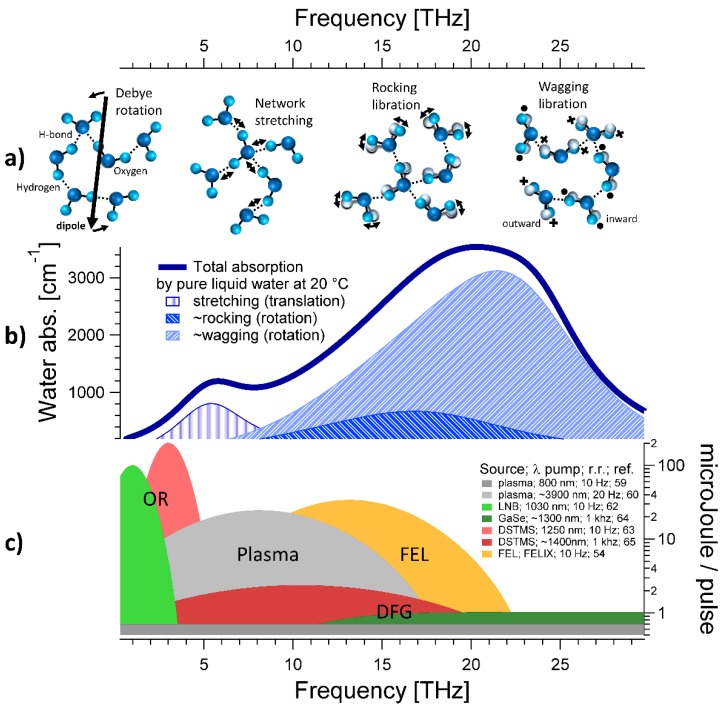
(**a**) Cartoons of the different modes of liquid water in the TetraHertz (THz) range. Below 1 THz, the dielectric response is dominated by the Debye-like reorientation of the collective dipole of H-bonded water molecules [18,19,20,21,22]. Intermolecular translations or “network stretching” modes are centered at ca. 6 THz, while librational modes are found between ~10 and ~20 THz [24,25,26]. (**b**) Equilibrium absorption coefficient of pure water at T = 20 °C [1,2,3,4]. A minimal fit reveals at least three bands that can be associated to the intermolecular modes sketched in panel (**a**). (**c**) Summary of the most intense THz sources reported to date. Gray: plasma generation; green: emission from inorganic crystals by non-linear optical methods; red: emission from organic materials; orange: frequency region of a pump-probe experiment operating at a free electron laser. The laser wavelength, repetition rate (r.r.), and the reference number is indicated. See text for further details.

While infrared spectroscopy (IR) typically probes intramolecular modes, the absorption features in the THz range can be broad [16]. These broad bands reveal properties related to the hydration of small or large solutes, including proteins [17]. Previous molecular dynamics (MD) calculations [1] indicated that different excitations are present in the IR (>30 THz) and THz ranges (<30 THz). While the intramolecular modes in the IR involve strong electronic couplings, the THz bands are linked to correlated intermolecular motions. MD also found that the water absorption band centered at ~6 THz can be described by dynamics extending to the first solvation shell. Below 3 THz, the collective nature and delocalized character of these low-frequency modes dominates, which involve systematic correlations of particle motion beyond the first solvation shell. Below 0.2 THz, the dielectric response of water is dominated by a band centered at ~20 GHz [18]. This band can be fitted to a Debye relaxation with a reorientation time of ~8 ps. While the original Debye model holds for non-interacting molecules exposed to an external electric field, the precise description of this band implies nontrivial microscopic mechanisms in the hydrogen-bonded network. Understanding this dielectric response of water is still a challenge above 20 GHz, up to the THz range [19,20,21,22]. The Debye-like reorientation mode is sketched on the left in Figure 1a.

At equilibrium, the absorption by liquid water in the THz spectrum is continuous and characterized by two prominent bands [23] that have been assigned to the hindered translational (centered at ~6 THz) and rotational (from ca. 10 to 20 THz) modes of the hydrogen-bonded water molecules [1,16]. The THz spectrum of pure liquid water at T = 20 °C is shown in Figure 1. In order to fit the band centered at ~20 THz shown in Figure 1b, at least two hindered rotational (or librational) modes are needed [24,25,26]. The infrared active rotations of a single water molecule have been assigned to the “rocking” and the “wagging”, while the “twisting” should be exclusively active in Raman experiments [26]. For this reason, and based on the different rotational constants, it is possible to tentatively assign the band centered at ~15 THz to the rocking librations of H-bonded waters. The band at ~21 THz can be associated to wagging librations. Cartoons of THz modes are sketched in Figure 1a.

In order to explore the complex energy landscape of liquid water and address its unique solvation properties, highly brilliant THz sources are required. Unfortunately, it is challenging to generate intense radiation in the so-called “THz gap”, which nowadays spans between about 7 and 19 THz and largely overlaps with water librations. We report in Figure 1c, to the best of our knowledge, the few available sources delivering pulsed radiation exceeding one µJ/pulse in the THz range.

There are two ways to generate intense THz pulses [27]. The first exploits current effects, i.e., the fact that a time-varying charge current can act as a radiation source [27]. This basic concept holds for antennas [28,29,30,31,32,33], which are widely used in conjunction with non-amplified pulsed lasers to perform terahertz time-domain spectroscopy, and for particle accelerators, where relativistic electron beams are deflected by magnets and thereby emit radiation of tunable wavelength [34,35,36] that can cover the THz range [37,38,39,40,41,42,43,44,45,46,47,48,49,50,51,52]. While accelerators can cover a large frequency range, these complex machines are often optimized for a certain range because optical elements have limited bandwidths [53]. For example, as sketched in orange in Figure 1c, single color pump-probe experiments at the free electron laser (FEL) facility FELIX in Nijmegen can currently be performed only between ca. 5 and 25 THz [54]. The severe beam time restrictions at the few available facilities, and the limited tunability in polarization and pulse length constrain the applicability of these sources.

Furthermore, THz generation is reported on using novel spintronic devices [55,56] as well as the broadband THz emission by plasma filaments [57,58]. Typically, THz emission by filaments is triggered by focusing into air the first and second harmonics of a fundamental near-infrared (NIR) amplified laser [57,58]. Dey et al. [59] demonstrated enhanced broadband intense THz generation by filamentation in liquids, with about 1 µJ/pulse/THz and spanning from ~0.1 to 50 THz (see the dark gray plot in Figure 1c. Recently, Koulouklidis et al. [60] proposed an extremely brilliant THz source by two color filamentation of MIR laser pulses with the fundamental wavelength set to 3.9 μm. Conversion efficiency above 2% and electric fields of more than 100 MV/cm were demonstrated spanning between ca. 3 and 11 THz. We sketch the corresponding spectrum with the light gray curve in Figure 1c.

Another way to generate THz is based on non-linear optical methods, by which a strong laser (often in the NIR) induces a non-linear polarization in a birefringent medium. In turn, the non-linear polarization can be the source of pulsed radiation [61]. Emission in the THz range can be achieved through optical rectification (OR) [62,63], by which the wavelength bandwidth of an infrared laser pulse is converted into an intrinsically phase-stable THz field, or by the difference frequency (DFG) [64,65] of two NIR pulses oscillating at slightly different frequencies. OR typically allows us to generate intense THz radiation below 5 THz (sketched in light green and light red in Figure 1c), while DFG covers higher frequencies (dark green and dark red in Figure 1c). Both inorganic (LiNbO_3_ [66,67,68,69,70,71,72,73,74,75,76,77], GaSe [78,79,80], ZnTe [81,82], LiGaS_2_ [83], GaP [84,85,86,87,88,89]) as well as organic (DSTMS [89,90,91,92], OH1 [93,94,95,96,97], DAST [98,99], DPFO [100], HMQ-TMS [101], OHQ-N2S [102]) crystals are phase-matched in the NIR and can emit pulsed THz fields with peak amplitudes in excess of 1 MV/cm. The approximate spectrum of the strongest THz pulses generated to date by non-linear optical methods in inorganic [62,64] and organic [63,65] crystals are shown in green and in red in Figure 1c, respectively.

The detection of THz radiation is based on similar concepts. The highest dynamic range (DR) reported to date is above 100 dbm when antennas are used in conjunction with multi-MHz laser oscillators [103,104]. For amplified NIR sources operating in the kHz range, we recently demonstrated [29] a fast scan acquisition technique based on the Pockels effect [37] with a DR of more than 60 dbm. Both techniques operate in a limited frequency range and can detect THz radiation up to ~7 THz. Broader THz spectra can be measured with thin crystals [89,105], via more exotic detection schemes employing organic crystals with large linear birefringence [106,107,108,109,110], or with air-biased coherent detection (ABCD) at a cost of reduced DR (~30 dbm) [111,112,113]. While the detection techniques described above allow measuring both the amplitude as well as the phase of a THz field, power or intensity detectors are also available over the entire THz range [114,115].

In the quest to find intense sources operating in the THz gap, here, we report the experimental characterization of the OR in GaP, OH1, and DSTMS over the full range of the wavelengths emitted by an optical parametric amplifier (OPA), i.e., between 1200 and 2500 nm.

## 2. Materials and Methods

An amplified Ti:Sa laser (Astrella, Coherent, Santa Clara, CA, USA) emits 90 fs long pulses centered at 790 nm with a repetition rate of 1 kHz. Part of the fundamental laser output (ca 2 mJ/pulse) seeds a commercial and automated OPA (TOPAS TWINS, Light Conversion). The non-linear process of parametric amplification generates simultaneously both the signal (s) as well as the idler (i) beams [61]. The total output power of the OPA (s+i) is between 0.6 and 0.8 W for the investigated wavelengths. We separate the s and i beams with a polarizing beam-splitter provided by the manufacturer. We use only one “pump” beam at a time, either s or i, to generate THz radiation. The selected pump pulse is focused down to a ~1 mm full width half maximum (FWHM) spot size into the crystal used to generate THz. We chop the pump beam at 500 Hz and adjust the pump fluence with neutral density filters. We keep the pump fluence well below the damage threshold, and set it to obtain peak THz fields of approximately 100 kV/cm.

Here we investigate the THz emission by ~0.5 mm thick crystals (GaP, DSTMS, and OH1) triggered by pump pulses centered at λ_P_ = 1200 (s), 1400 (s), 1600 (s), 2100 (i), 2300 (i), 2500 nm (i). We chose the wavelengths to avoid photodamage. OH1 [116] (DSTMS [117,118]) strongly absorbs at 1500, 1800, and 2000 nm (1700 and 1800 nm). We recorded the generated THz fields by electro-optical sampling [32,37,81,119,120,121] in another 0.5 mm thick GaP crystal. The emitted field is probed by a 790 nm laser beam detected by balanced photo diodes (PDB210A, Thorlabs, Newton, NJ, USA) and lock-in amplification with a boxcar averager (UHFLI, Zurich Instruments, Zurich, Switzerland). The photovoltage is converted into field amplitude (kV/cm) by considering the electro-optical coefficient, index of refraction, and thickness of the detection crystal (see e.g., Equation (17) in [37]). We enclose the setup into a plastic box and purge with nitrogen to reduce the water vapor content to below 10%. 

## 3. Results

**Figure 2:**
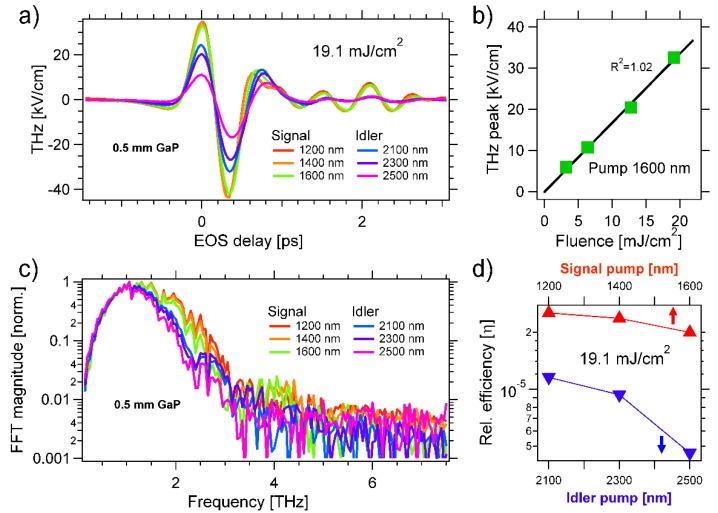
(**a**) Phase-resolved THz fields emitted by optical rectification in 0.5 mm thick GaP crystal. We use as pump pulses the output of an optical parametric amplifier between 1200 and 2500 nm. The pump fluence is set to 19.1 mJ/cm^2^ for all pump wavelengths (λ_P_). The THz is detected by electro-optical sampling (EOS) in another 0.5 mm thick gallium phosphide (GaP) crystal. We use an additional 90 fs long sampling beam at 790 nm for EOS. (**b**) We set the pump to λ_P_ = 1600 nm and plot the peak THz field value at t_EOS_ = 0 ps versus pump fluence. We obtain a linear fit with an R^2^ value of 1.02, which confirms THz emission by a nonlinear optical process of rectification. (**c**) Normalized spectrum of the THz field as a function of λ_P_. As expected, the frequency range is increased for shorter wavelengths [84], it is broad at λ_P_ = 1200 nm and narrow at λ_P_ = 2500 nm. (**d**) Estimated conversion efficiency of NIR radiation into THz radiation as a function of pump color. The efficiency is η~3 × 10^−5^ at 1200 nm and decreases for larger λ_P_.

**Figure 3:**
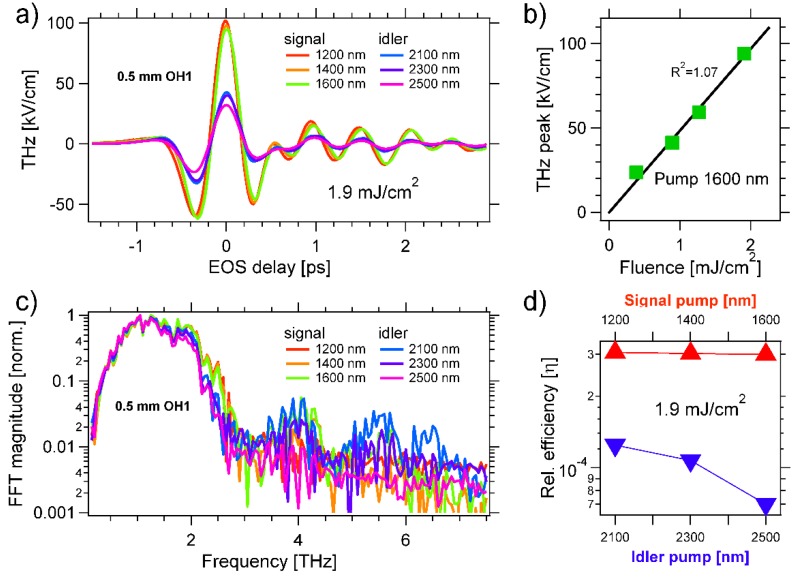
(**a**) Almost single-cycle THz fields emitted by optical rectification (OR) of optical parametric amplifier (OPA) pulses in a ~0.5 mm thick OH1 crystal. The pump fluence is set to 1.9 mJ/cm^2^. The THz is detected by EOS in a 0.5 mm thick GaP crystal. For EOS, we use an additional 90 fs sampling beam at 790 nm and chop the pump at 500 Hz. (**b**) We set the pump to λ_P_ = 1600 nm and detect the peak THz field at t_EOS_ = 0 ps versus pump fluence. A linear fit with an R^2^ value of 1.07 is obtained, confirming that THz is emitted by an OR process. (**c**) Normalized spectrum of the THz field emitted as a function of λ_P_. The spectrum has high-frequency components around ca. 4 THz and between 5 and 7 THz, that are enhanced at λ_P_ = 2100 nm. (**d**) Estimated conversion efficiency of infrared into THz radiation by OH1 as a function of λ_P_. The efficiency is η~3 × 10^−4^ between 1200 and 1600 nm, and is smaller when pumped by the idler.

**Figure 4:**
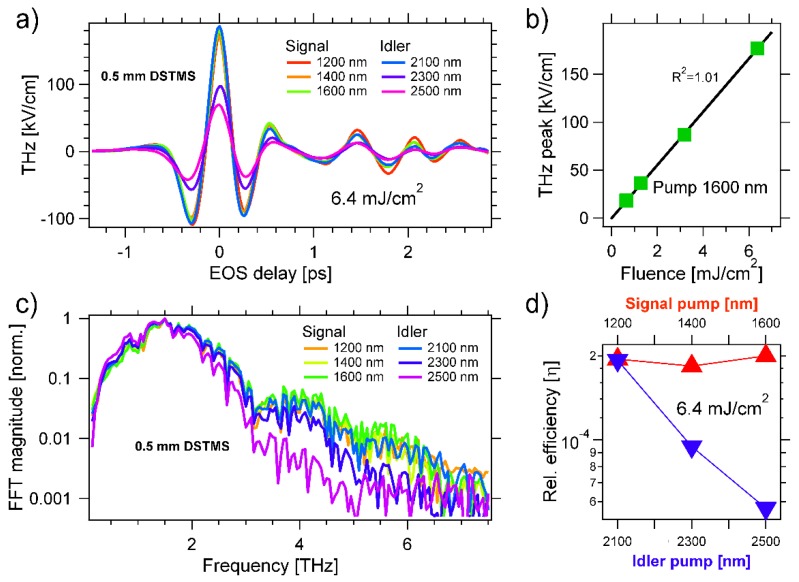
(**a**) THz emission by a ~0.5 mm thick 4-N,N-dimethylamino-4′-N′-methyl-stilbazolium 2,4,6-trimethylbenzenesulfonate (DSTMS) crystal. The pump fluence is set to 6.4 mJ/cm^2^. The THz is detected by EOS, see text for details. (**b**) We set the pump to λ_P_ = 1600 nm, vary the pump fluence, and detect the peak THz field at t_EOS_ = 0 ps. We obtain a linear trend that can be fitted with an R^2^ value of 1.01. (**c**) Normalized spectrum of the THz field generated for different pump wavelengths. The spectrum is broader in DSTMS than in either GaP (Figure 2) or OH1 (Figure 3), with high frequency components extending over the entire detectable region [126]. (**d**) Estimated efficiency of the THz generation in DSTMS pumped in the NIR. The efficiency, largest between 1200 and 2100 nm, and amounts to ca. η~2 × 10^−4^.

In the following, we report the THz emission by both the inorganic GaP as well as the organic DSTMS and OH1 crystals (Rainbow Photonics, Zurich, Switzerland) at the OPA wavelengths. The results are summarized in Figure 2, Figure 3 and Figure 4, displaying the THz emission by ~0.5 mm thick GaP, OH1 and DSTMS, respectively.

### 3.1. Gallium Phosphide (GaP)

Figure 2a displays the THz fields generated for different pump wavelengths, all obtained at the same pump fluence of 19.1 mJ/cm^2^. The transient THz pulses are detected by delaying the arrival time of the electro-optical sampling beam (t_EOS_). We obtain almost single-cycle fields with a duration of ca. 0.35 ps (time difference between the minimum at t_EOS_ ~ −0.15 ps and the next minimum at t_EOS_ ~ +0.2 ps). This duration increases for longer pump wavelengths. A THz peak field of ~30 kV/cm is obtained at t_EOS_ = 0 ps and for λ_P_ = 1200, 1400, and 1600 nm. The THz field amplitude emitted by the non-linear optical process of OR should scale linearly with the intensity of the driving pump field [61], which is confirmed in Figure 2b. We chose the intermediate pump wavelength of λ_P_ = 1600 nm and varied the fluence from 3.2 mJ/cm^2^ to 19.1 mJ/cm^2^. We found a linear relationship between the THz field and the pump intensity, as indicated by the black line fit with an R^2^ value of 1.02.

Figure 2c displays the magnitude of the Fourier transformation (FFT) of the THz fields displayed in Figure 2a. In order to compare the different frequency components, the maximum magnitude value is normalized to one in each spectrum. We found that most of the THz radiation is emitted between 0.5 and 2.5 THz. Shorter pump wavelengths yield emission of broader THz spectra. In Figure 2d, we report the relative efficiency (η) of the energy conversion of NIR into THz radiation. We estimated this efficiency to the ratio of the THz fluence and the pump fluence. The pump fluence of 19.1 mJ/cm^2^ was obtained from the pump spot size at the generation crystal (~1 mm FWHM) and the input pump power, measured with a power meter (FieldMaxII-TO PM10, Coherent). We calculated the THz fluence from the THz peak field (measured with electro-optical sampling (EOS)) and the THz spot size at the detection crystal (estimated to ~0.4 mm FWHM with an iris). For the 0.5 mm thick GaP, we found that η decreases for increasing pump wavelength, η~3 × 10^−5^ at λ_P_ = 1200 nm and η~5 × 10^−6^ at λ_P_ = 2500 nm (see Figure 2d). Please note that the value of the relative efficiency is only valid under ideal conditions. In fact, this efficiency does not include reflection losses nor the nontrivial contributions by both the wavelength-dependent pump absorption in the different generation crystals [116,117,118,122,123] as well as the response function of the detecting apparatus [124,125,126].

### 3.2. 2-{3-(4-hydroxystyryl)-5,5-dimethylcyclohex-2-enylidene}malononitrile (OH1)

Figure 3a displays the THz fields generated for different pump wavelengths, all obtained at the same pump fluence of 1.9 mJ/cm^2^ to avoid damaging the ~0.5 mm thick OH1 crystal. In all cases, we obtained almost single-cycle fields with a duration of ca. 0.3 ps (time difference between the minimum at t_EOS_ ~ −0.15 ps and the next minimum at t_EOS_ ~ +0.15 ps). A THz peak field of ~100 kV/cm is obtained at t_EOS_ = 0 ps and for λ_P_ = 1200, 1400, and 1600 nm. In Figure 3b, we show the linear dependence between the THz field amplitude and the pump fluence. We set the pump wavelength to λ_P_ = 1600 nm and varied the fluence from 0.4 mJ/cm^2^ to 1.9 mJ/cm^2^. As proved by the black line fit (R^2^ = 1.07), a linear relationship held. Figure 3c displays the normalized magnitude of the FFT of the THz fields displayed in Figure 3a. While most of the THz radiation was emitted between 0.5 and 2 THz, additional sub-bands were found around ca. 4 THz and between 5 and 7 THz. These high frequency components were enhanced at λ_P_ = 2100 nm. In Figure 3d, we report the relative efficiency of the NIR-to-THz energy conversion by OH1. We found η~3 × 10^−4^ for λ_P_ = 1200 nm, 1400 nm, 1600 nm. The efficiency dropped at longer wavelengths.

### 3.3. 4-N,N-dimethylamino-4′-N′-methyl-stilbazolium 2,4,6-trimethylbenzenesulfonate (DSTMS)

Figure 4a displays the THz fields generated via OR in DSTMS, all obtained at the same pump fluence (6.4 mJ/cm^2^). For most pump wavelengths, we obtained single-cycle fields with a duration of ca. 0.27 ps (the first minimum is at t_EOS_ ~ −0.15 ps and the second at t_EOS_ ~ +0.12 ps). A THz peak field of ~177 kV/cm was obtained at t_EOS_ = 0 ps for λ_P_ = 1200 nm, 1400 nm, 1600 nm, and 2100 nm. We chose the pump wavelength of λ_P_ = 1600 nm and varied the fluence from 0.6 mJ/cm^2^ to 6.4 mJ/cm^2^. The result is plotted in Figure 4b which confirms a linear relationship is found between the emitted THz field and the pump intensity, as demonstrated by the black line fit (R^2^ = 1.01). Figure 4c displays the normalized magnitude of the FFT of the THz fields displayed in Figure 4a. We found that DSTMS emits THz radiation over a broad frequency range that spans the entire acquisition window from ca. 0.5 to 7 THz. The bandwidth of the emission spectrum was smaller at longer pump wavelengths. In Figure 4d, we report the relative efficiency of the OR nonlinear process in DSTMS. We found η~ 2 × 10^−4^ for λ_P_ = 1200, 1400, 1600, and 2100 nm. The efficiency dropped for the larger wavelengths and amounts to η~6 × 10^−5^ at λ_P_ = 2500 nm.

## 4. Discussion

Previously, we investigated the THz emission by GaP pumped by a mode-locked oscillator tunable between 700 and 1000 nm [84]. We found that the THz emission covers a broad range at λ_P_ = 900 nm, and that the frequency range of the emitted spectrum decreases for longer pump wavelengths. The results reported in Figure 2 corroborate these earlier findings (see Figure 2c). This benchmark confirms the validity of our experimental investigation. As expected, we also found that both organic crystals are more efficient THz sources than gallium phosphide. The relative energy conversion efficiencies of GaP, OH1, and DSTMS at λ_P_ = 1600 nm are η~2 × 10^−5^, η~3 × 10^−4^, and η~2 × 10^−4^, respectively. 

In order to perform non-linear THz experiments on liquid water (Figure 1), broad and intense THz sources are required. The pump intensity required to drive liquid water into the nonlinear response regime depends on the wavelength. Based on the third-order responses reported previously, we tentatively estimate to 50 GW/cm^2^, 5 TW/cm^2^, 1 GW/cm^2^, and 5 TW/cm^2^, the peak power required to induce a pump-probe signal of roughly 1% at the frequency of ~1 MHz [127,128], ~1 THz [72], ~10 THz [54], and ~200 THz [129,130,131,132,133,134,135,136,137,138,139,140], respectively.

With the aim of generating such intense pump fields, here, we investigated the THz emission by organic crystals with high electro-optic coefficients and pumped by laser pulses spanning the entire OPA spectrum. Peak fields above 0.1 MV/cm were emitted by both OH1 (Figure 3) as well as DSTMS (Figure 4) for loosely focused pump pulses and fluences of only few mJ/cm^2^. Both organic crystals also emit some radiation over the whole detection range and up to 7 THz [126], with DSTMS emitting the broadest THz spectra (Figure 4). To the best of our knowledge, here, we reported—for the first time—the THz generation by OR in OH1 and DSTMS pumped by idler beams at the wavelength λ_P_ ≥ 2100 nm. As shown in Figure 4, we found out that DSTMS emits a broad spectrum at λ_P_ = 2100 nm with an unchanged high efficiency (η~2 × 10^−4^). Thus, this crystal could generate intense THz radiation from special laser sources operating in the NIR.

Future experimental developments should aim both at scaling up the pump fluence without damaging these fragile organic crystals, as well as extending the frequency bandwidth of the detection apparatus without losing dynamical range. Pulse-shaping techniques [89,141] controlling the radiation wavefront [71,94] and/or the spatiotemporal chirp [65] of the laser beams could possibly be optimized to broaden the THz spectrum.
